# Supplementary material for: Faricimab Reverts VEGF-A165-Induced Impairment of the Barrier Formed by Retinal Endothelial Cells
Source: Int J Mol Sci. 2025 May 1;26(9):4318. doi: 10.3390/ijms26094318 (PMC12072733; doi:10.3390/ijms26094318)

Supplementary material to:

Faricimab reverts VEGF-A_{165}-induced impairment of the barrier formed by retinal endothelial cells

Dominik M. Jung, Isabell Fuezy, Lyubomyr Lytvynchuk, Heidrun L. Deissler

General Information on supplementary Figures S1-S3, Original images of Western-blot analyses:

To determine presence of multiple antigens in parallel, the protein-bound membrane was cut in two or three pieces (see following pages) and exposed to the mentioned antibodies. Afterwards the membrane parts were incubated for 45 min in Restore Plus Western Blot Stripping Buffer (ThermoFisher Scientific) at room temperature to remove bound antibodies. After washing three times for 5 min with 0.1% Tween-20/PBSd (phosphate-buffered saline without Ca^{2+} and Mg^{2+}) and blocking, the membrane parts were exposed to another set of antibodies. This procedure was followed for a second time, if necessary.

Chemiluminescence signals were always directly scanned as described in section "4.5 Western blot analyses of protein extracts"; depending on signal intensity, several images with different exposure times were taken. A black/white or color image of the membrane containing the prestained size marker proteins was taken in parallel and merged with the corresponding image of the membrane containing antibody-specific signals by the EvolutionCapt edge software (Version 18.12; Vilbert Lourmat). Labels were added to the merged image using the editing-function of the software.

Supplementary Figure S1:

1. Cell extracts were prepared from iBREC exposed to VEGF-A_{165} for **one** day.
2. Proteins were separated by SDS-PAGE and transferred to a PVDF-membrane. After blocking of the membrane, it was cut into three pieces below 35 kDa and above 55 kDa.
3. The upper part (55 kDa-250 kDa) was exposed to antibodies against VEcadherin, the middle part (35 kDa-55 kDa) against actin and the lower part (<35 kDa) against claudin-1.

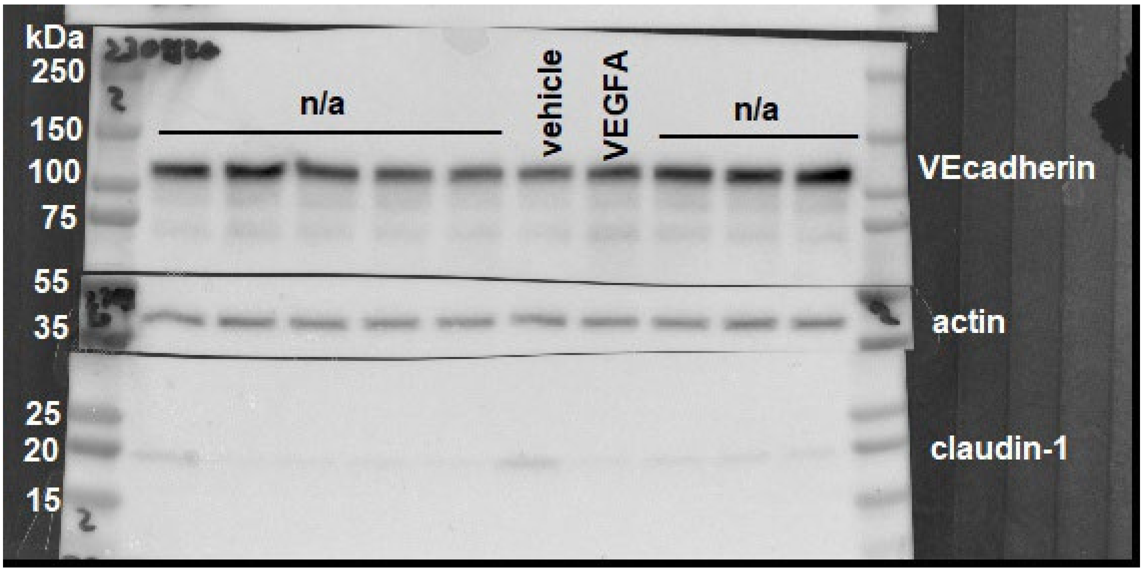

4. Lower part (<35 kDa) of the membrane was washed in Restore Plus Western Blot Stripping buffer at room temperature for 45 min to remove bound antibodies. After blocking, it was exposed to claudin-5-specific antibodies.

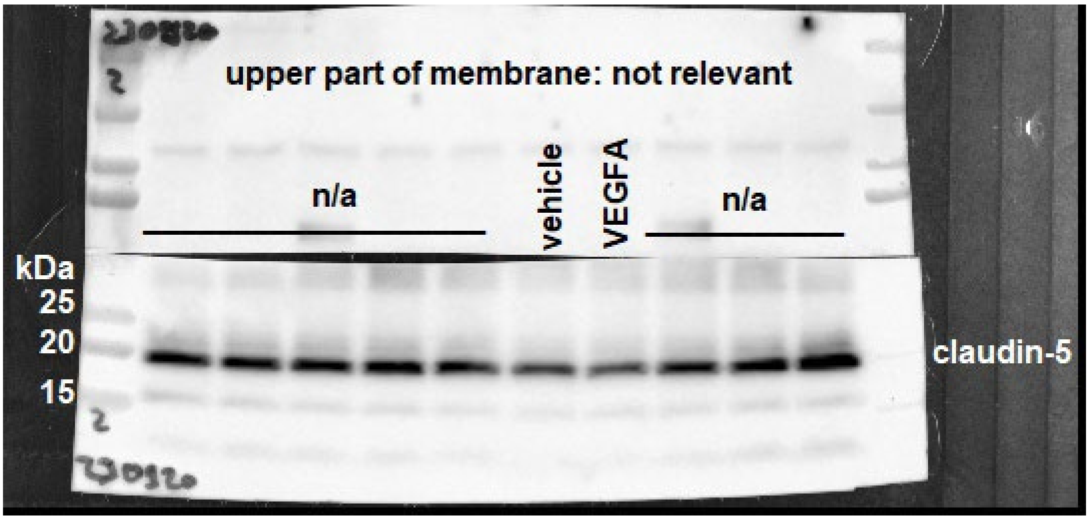

Supplementary Figure S2:

1. Cell extracts were prepared from iBREC exposed to VEGF-A165 for **one** day and subsequently to faricimab for **two** days.
2. Proteins were separated by SDS-PAGE and transferred to a PVDF-membrane. After blocking of the membrane, it was cut into two pieces below 35 kDa.
3. The upper part (35 kDa-250 kDa) was exposed to antibodies against PLVAP, the lower part (<35 kDa) against claudin-1.

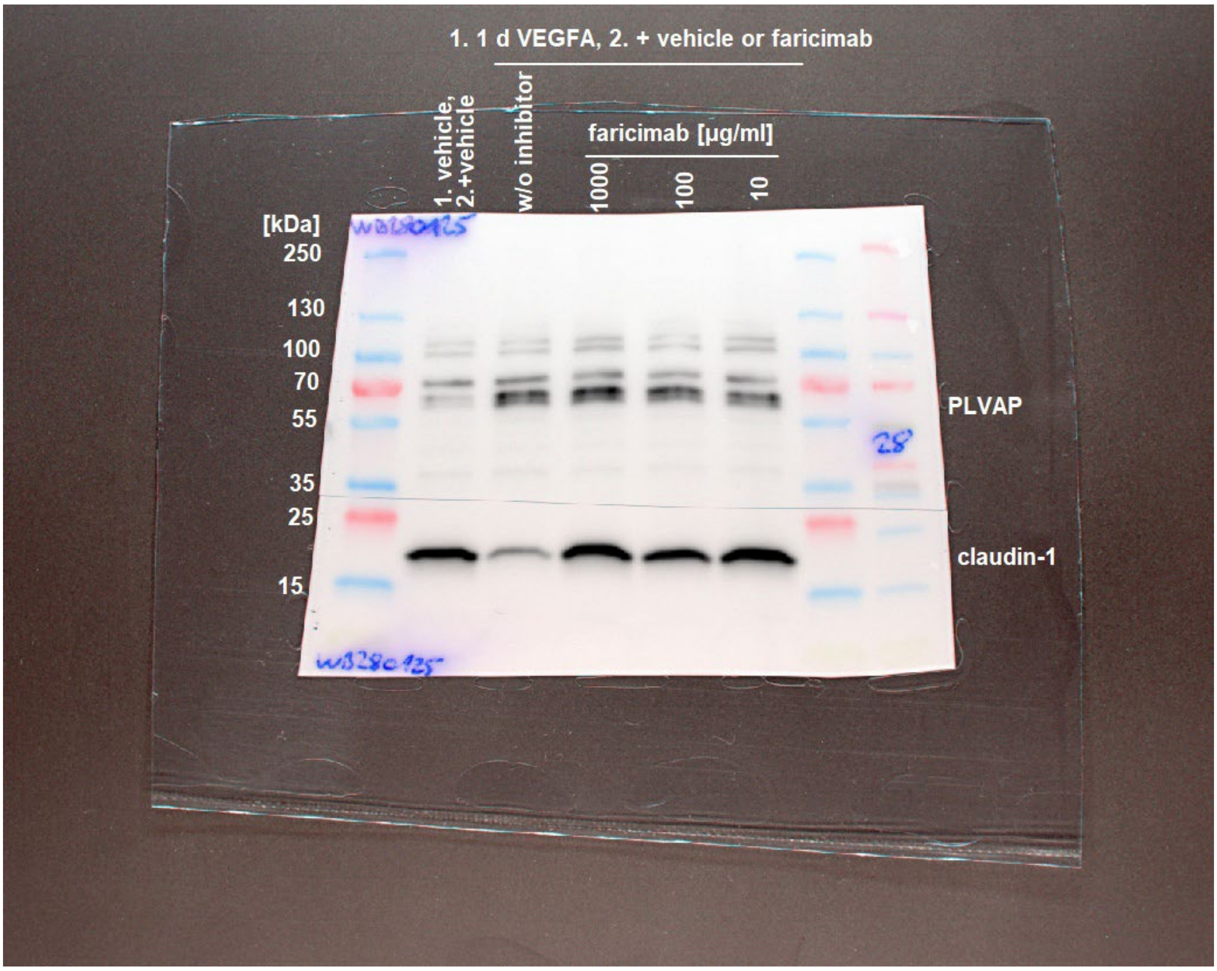

4. After exposure, both parts of the membrane were washed in Restore Plus Western Blot Stripping buffer at room temperature for 45 min to remove bound antibodies. After blocking, the upper half of the membrane was cut into two pieces above 70 kDa.
5. Parts of the membrane were incubated with antibodies against VEcadherin (70-250 kDa), β-actin (35-70 kDa), or claudin-5 (<35 kDa).

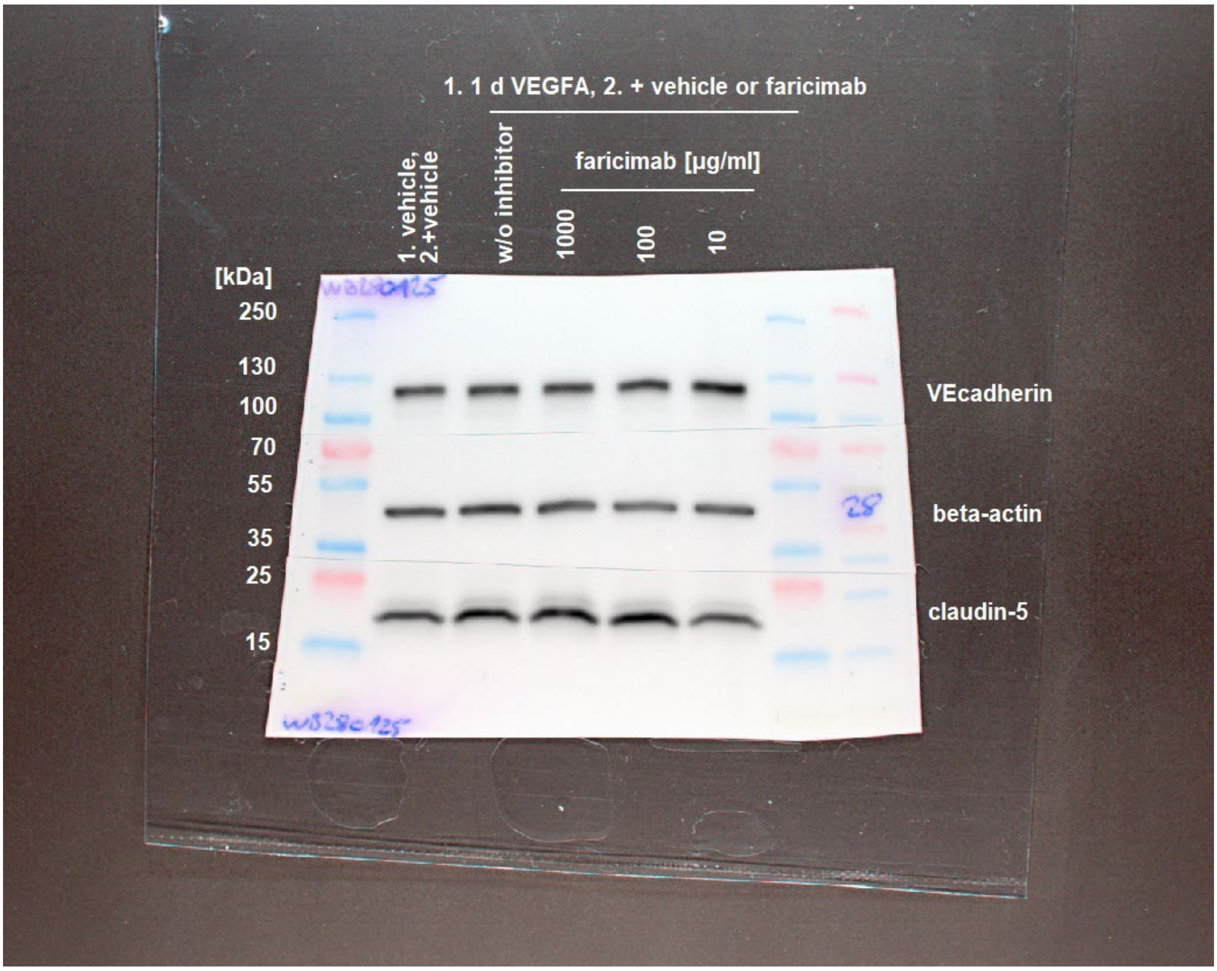
1. 1 d VEGFA, 2. + vehicle or faricimab
1. vehicle, 2.+vehicle
w/o inhibitor
faricimab [µg/ml]
1000
100
10
[kDa]
250
130
100
70
55
35
25
15
VEcadherin
beta-actin
claudin-5

Supplementary Figure S3:

1. Cell extracts were prepared from iBREC exposed to VEGF-A_{165} for for **one** day and subsequently to faricimab for **five** days.
2. Proteins were separated by SDS-PAGE and transferred to a PVDF-membrane. After blocking of the membrane, it was cut into two pieces below 35 kDa.
3. The upper part (35 kDa-250 kDa) was exposed to antibodies against PLVAP, the lower part (<35 kDa) against claudin-1.

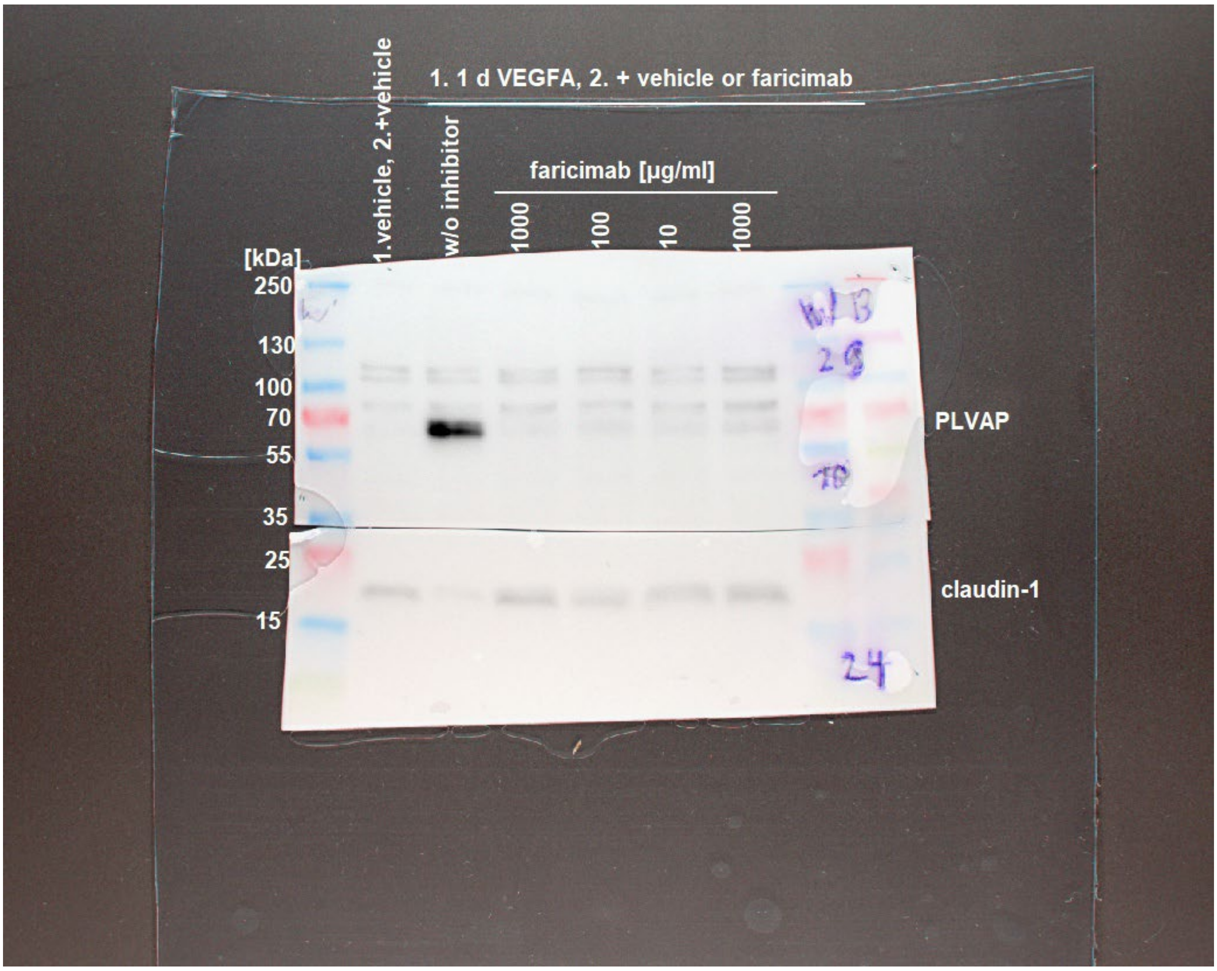

4. Both parts of the membrane were washed in Restore Plus Western Blot Stripping buffer at room temperature for 45 min to remove bound antibodies. After blocking, the upper half of the membrane was cut into two pieces above 70 kDa.
5. Parts of the membrane were incubated with antibodies against VEcadherin (70-250 kDa), β-actin (35-70 kDa) or claudin-5 (<35 kDa).

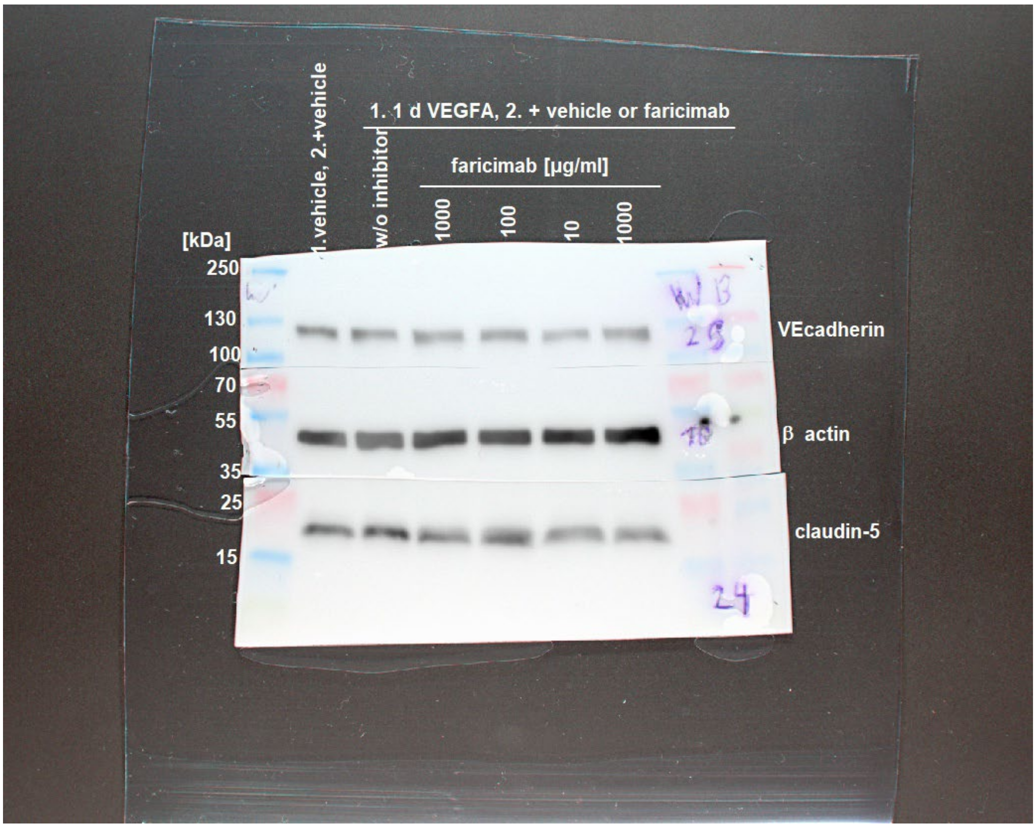

6. Middle part (35-70 kDa) of the membrane was again washed in Restore Plus Western Blot Stripping buffer at room temperature for 45 min to remove bound antibodies. After blocking, it was incubated with antibodies against human IgG γ chains to assess amounts of internalized faricimab.

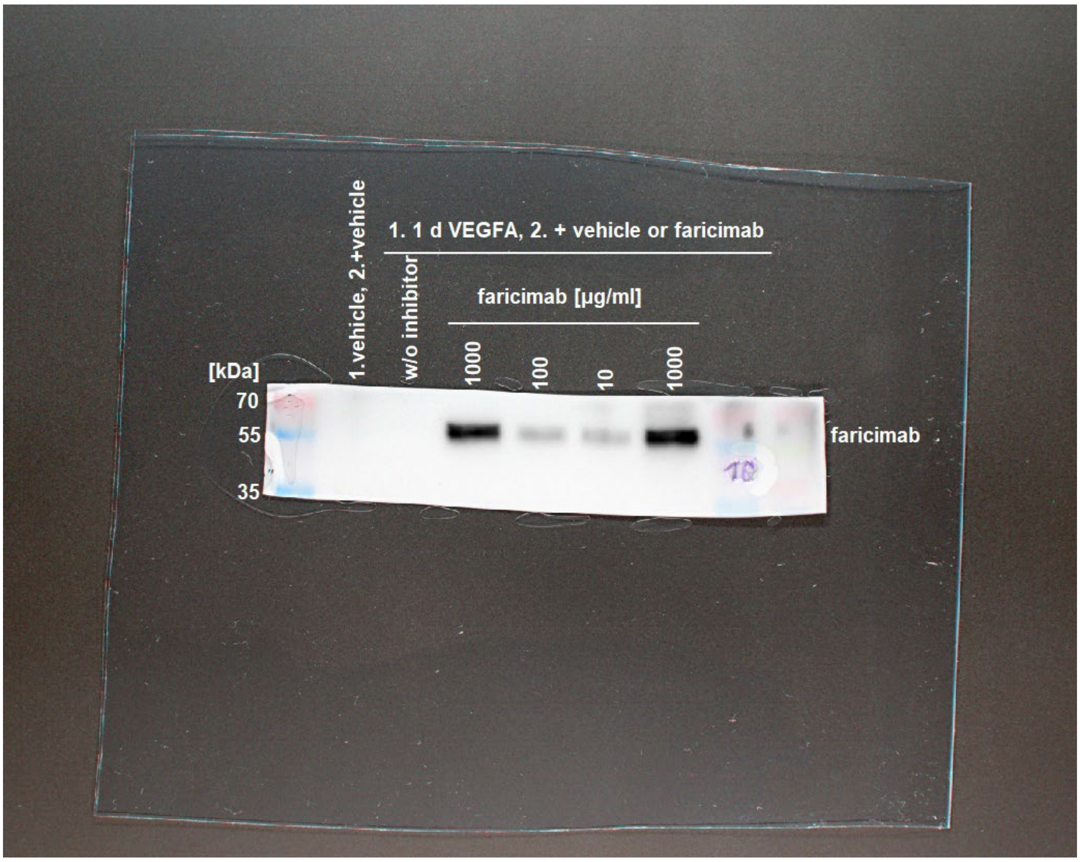

Supplement: Supplementary file 1 [file ijms-26-04318-s001.zip › ijms-3549685-supplementary.pdf]
